# Does the Time of Day at Which Endocrine Therapy Is Taken Affect Breast Cancer Patient Outcomes?

**DOI:** 10.3390/curroncol28040229

**Published:** 2021-07-06

**Authors:** Ana-Alicia Beltran-Bless, Lisa Vandermeer, Mohammed F. K. Ibrahim, Brian Hutton, Risa Shorr, Marie-France Savard, Mark Clemons

**Affiliations:** 1 Division of Medical Oncology, Department of Medicine, University of Ottawa and the Ottawa Hospital, Ottawa, ON K1H 8L6, Canada; abeltran@toh.ca (A.-A.B.-B.); msavard@toh.ca (M.-F.S.); 2 Ottawa Hospital Research Institute, Ottawa, ON K1Y 4E9, Canada; lvandermeer@ohri.ca (L.V.); bhutton@ohri.ca (B.H.); 3 Division of Clinical Sciences, Medical Oncology, Northern Ontario School of Medicine, Thunder Bay, ON P7B 5E1, Canada; ibrahimm@tbh.net; 4 School of Epidemiology, Public Health and Preventive Medicine, University of Ottawa, Ottawa, ON K1G 5Z3, Canada; 5 Ottawa Hospital, Ottawa, ON K1H 8L6, Canada; rshorr@toh.ca

**Keywords:** tamoxifen, aromatase inhibitor, breast cancer, chronotherapy, side effects

## Abstract

Background: Non-compliance and non-persistence with endocrine therapy for breast cancer is common and usually related to treatment-induced side effects. There are anecdotal reports that simply changing the time of day when taking endocrine therapy (i.e., changing morning dosing to evening dosing or vice versa) can reduce side effects. Literature review: We conducted a literature review to evaluate whether changing the timing of tamoxifen and/or aromatase inhibitor administration impacted patient outcomes. No randomized control trials or prospective cohort studies that looked at time of day of endocrine therapy were identified through our review of literature from 1947 until August 2020. Conclusions: Given the rates of endocrine therapy non-compliance and non-persistence reported in the literature, ranging from 41–72% and 31–73%, respectively, simply changing the time of day when medications are taken could be an important strategy. We could identify no trials evaluating the effect of changes in timing of administration of endocrine therapy on breast cancer patient outcomes. Randomized control trials are clearly indicated for this simple and cost-effective intervention.

## 1. Introduction

Endocrine therapy is a standard of treatment for hormone receptor-positive breast cancer [1,2]. The side effects of endocrine therapy are well-recognized and can lead to treatment non-compliance (i.e., extent of adherence to the recommended administration schedule) or non-persistence (i.e., not continuing treatment for the prescribed duration) [3,4]. A systematic review of adjuvant endocrine treatment found compliance ranged from 41–72% and persistence from 31–73% [5]. Treatment adherence is especially important in breast cancer, as non-persistence and reduced compliance to endocrine therapy have been associated with worse disease outcomes [6]. While many different lifestyle and pharmacologic interventions have been used to mitigate treatment side effects, there is anecdotal evidence from health care providers and patients themselves regarding changing the time of day of their endocrine therapy (from morning to evening administration or vice versa), with narratives of success [7]. 

Circadian rhythms, the internal processes that regulate cell metabolism and energy consumption over a 24 h schedule, are believed to play a direct role in drug effectiveness and toxicity [8]. Indeed, chronotherapy, or adjusting dosing of medication to a specific time of the circadian cycle, is not a new concept; however, few dedicated studies exist. There is evidence suggesting that the adherence to drugs, such as oral diabetes medications, might be better in the morning as compared to the evening [9]. Recently, a study showed that the time of day of antihypertensives could significantly impact their effectiveness [10]. In oncology, there is evidence that re-synchronizing circadian rhythms with bedtime melatonin can increase sleep duration and sleep quality, as well as improve quality of life and social/cognitive domains [11,12]. Some studies have been performed evaluating the use of chronotherapy to determine timing of chemotherapy in colorectal cancer and breast cancer and of oral tyrosine kinases in renal and gastrointestinal cancers [13,14,15,16,17,18]. Results were mixed, with some showing improvement in toxicities with chronomodulated treatments [13,14,15,16]. 

Breast cancer patients often seek insights on online forums to determine the optimal time of day to take their endocrine therapy. While some patients have made recommendations that night administration improves side effects [7], we are not aware of any appropriately designed studies evaluating this. It seems that changing the time of day of endocrine therapy is already something that is done in practice. However, there is a lack of relevant data on the subject, and no evidence of any real benefit has been established with regards to side effect profile, quality of life implications, or survival. To this end, we conducted a literature review to evaluate all currently available data. This information could be used to both better inform patients and to identify gaps in the literature that require further study.

## 2. Literature Review

A protocol was prepared a priori to guide the performance of this work and was registered with PROSPERO (Registration number CRD42020204822). The research question was phrased in the Population-Intervention-Comparator-Outcomes-study design framework as “Does the time of day at which endocrine therapy is taken affect outcomes in patients with breast cancer?” This review was prepared in consideration of guidance from the PRISMA statement [19,20]. 

We searched English language journal publications from Ovid MEDLINE^®^, Embase and Embase Classic, and Cochrane Register of Controlled Trials published from 1947 to August 2020. The search included terms related to breast cancer and timing of endocrine therapy. The full search strategy is outlined in a supplemental file (Supplemental Information 1) and was designed and implemented by an information specialist (RS). We also conducted a review of the U.S. National Library of Medicine ClinicalTrials.gov, which did not reveal any past or current clinical trials on this topic. 

**Figure 1:**
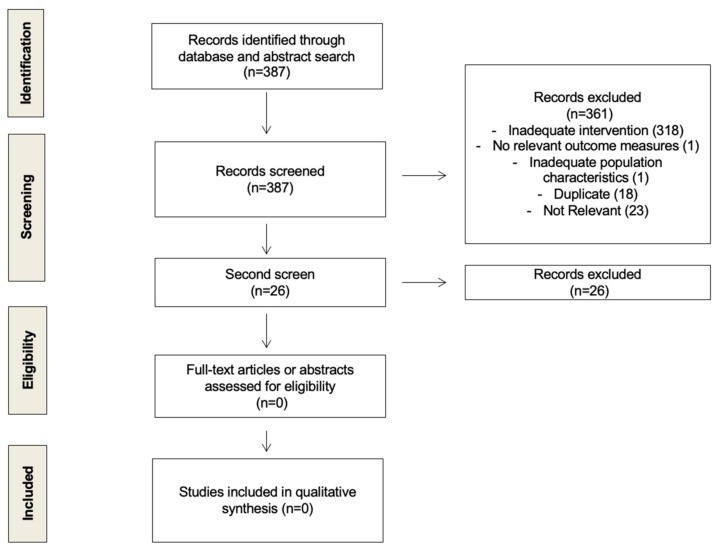
Flow diagram of study selection process.

We looked for randomized control trials and prospective cohort studies that examined the difference in the time of day (e.g., morning vs. evening) at which women received their endocrine therapy (tamoxifen or aromatase inhibitors) for estrogen/progesterone receptor-positive (ER/PR+) invasive breast cancer. After screening 361 unique citations, we did not find any that had previously looked at this question. We conducted a second screen adding another broad term, “circadian” (Supplemental Information 2). This process of study assessment was documented using a flow diagram (Figure 1). 

## 3. Discussion

While many strategies exist to reduce side effects and improve compliance and persistence with endocrine therapy for breast cancer, it appears that health care providers and patients themselves will often simply change the time of day that they take their medications, with some anecdotal evidence of success [7]. This fits with the underlying concept of chronotherapy, i.e., changing the time of day of treatment in order to directly influence drug effectiveness and toxicity [8]. Unfortunately, despite the prevalence of breast cancer globally and the frequent use of endocrine therapy, no studies were identified that have examined this question. 

There have been examples of chronobiology trials in medicine. The Hygia Chronotherapy trial found that bedtime administration of anti-hypertensive therapy led to better ambulatory blood pressure control and a decrease in primary cardiovascular outcomes [10]. This was felt to be partly linked to improved targeting of underlying circadian rhythm-organized biological mechanisms in cardiovascular health. The results of chronotherapy in oncology have been less clear. Between 1994 and 2005, three randomized clinical trials were conducted by Lévi and his team, looking at fluorouracil and oxaliplatin given as continuous infusion versus chronomodulated via a pharmacokinetic model. While all chronomodulated treatments reduced toxicity, survival benefits were mixed: one showed a benefit in median survival, while the others did not show any survival benefit; however, subgroup analysis of the most recent study revealed a three-month survival advantage only in men [13,14,15]. Improving circadian rhythm with bedtime melatonin has been shown to improve quality of life and sleep quality and duration in cancer patients, known for circadian dysrythmicity [12]. In metastatic breast cancer patients, a study looking at optimal circadian time of vinorelbine and 5-fluorouracil found that the least toxic time alternated for various toxicities [16]. The only study on timing of oral anti-cancer medication has been with sutent in metastatic renal cell and gastrointestinal stromal tumours. Both studies revealed some reduced grade 3 adverse events with morning administration, although this was not significant [17,18].

Developing new ways to mitigate side effects is important as we are increasingly prescribing longer durations of treatment [21,22] and because reduced compliance to endocrine treatment leads to reduced disease-free survival [6]. We also know that estrogens have a specific role in modulating circadian rhythms and that many cytochromes oscillate in a time-dependent manner [23,24]. A study looking at circadian variation of tamoxifen pharmacokinetics in humans and mice examined plasma exposure to tamoxifen and its metabolites with morning, afternoon, and evening administration, with cross over in 12 breast cancer patients. Results showed marginal differences in pharmacokinetic parameters between morning and evening administration, although they did find that systemic exposure (AUC0–24 h) to endoxifen was 15% higher following morning administration. While this increased exposure to endoxifen was felt to be compatible with more rapid absorption of lipophilic drugs in the morning, other hypotheses included a circadian variation in metabolism (through CYP3A4, CYP2D6, etc.). The study was not powered to detect potential differences in efficacy or side effects. Interestingly, women reported changes in incidence and severity of hot flashes with the different dose timing administrations, but details were not given [25]. 

It is possible that there were case reports and retrospective series evaluating the effects of time of drug administration; however, our methodology for this literature review was to only access prospective trials. We also checked clinicaltrials.gov to see if any studies were cited, as well as reviewing the reference sections of the previously published chronobiology studies [12,13,14,15,16]. None of these strategies identified any additional studies. 

In conclusion, while anecdotal evidence suggests that a trial-and-error approach for some patients of changing the time of day of taking their endocrine therapy may reduce side effects and improve quality of life in some patients, we were unable to identify any supporting studies. Given that many studies have been conducted investigating the use of more toxic and expensive interventions for reducing side effects, further studies evaluating this low cost, practical, and simple intervention are clearly warranted. A prospective pragmatic, multicentre, randomized clinical trial, REaCT-CHRONO, will soon start to establish the optimal timing (morning vs. evening) of administering endocrine therapy based on side effects and benefits in early-stage breast cancer patients (clinicaltrials.gov, NCT04864405) [26]. While the primary endpoint of this study will be endocrine toxicity and tolerability, other endpoints will include patient quality of life, non-persistence, or non-compliance with initially prescribed endocrine therapy, as well as cost-effectiveness. We believe this study of 235 patients will be the world’s first prospective randomized trial of optimal time-finding of endocrine therapy in breast cancer patients. 

## Supplementary Materials

The following are available online at https://www.mdpi.com/article/10.3390/curroncol28040229/s1. Figure S1. Search Strategy 1. Figure S2. Search Strategy 2.
