# Supplementary material for: Does the Time of Day at Which Endocrine Therapy Is Taken Affect Breast Cancer Patient Outcomes?
Source: Curr Oncol. 2021 Jul 6;28(4):2523–8. doi: 10.3390/curroncol28040229 (PMC8293101; doi:10.3390/curroncol28040229)
Supplement: Supplementary file 1 [file curroncol-28-00229-s001.zip › curroncol-1281526-supplementary/Supplemental Figures/Supplemental Figure S1.pdf]

Supplemental Figure S1: Search Strategy 1

Database: Embase Classic+Embase <1947 to 2020 August 26> , Ovid MEDLINE(R) ALL <1946 to August 26, 2020>, EBM Reviews - Cochrane Central Register of Controlled Trials <July 2020>
Search Strategy:
----

1 exp Breast Neoplasms/ (863076)
2 (breast adj2 (cancer or neoplasm* or tumo?r* or carcinoma*)).tw. (771998)
3 (breast and (cancer or neoplasm* or tumo?r* or carcinoma*)).kf. (59576)
4 or/1-3 (1032377)
5 exp Aromatase Inhibitors/ (42347)
6 Aromatase inhibitor*.tw,kw. (21929)
7 exp Tamoxifen/ (85879)
8 (tamoxifen or anastrozole or exemestane or Fadrozole or formestane or intraovarian peptides or letrozole or plomestane or vorozole).tw,kw. (74869)
9 Antineoplastic Agents, Hormonal/tu (10910)
10 ((hormon* or endocrin* or systemic) adj (treatment or therapy)).tw. (170669)
11 or/5-10 (287655)
12 4 and 11 (112785)
13 *Time Factors/ (3708)
14 "time of day".tw,kw. (25635)
15 time of administration.tw,kw. (8785)
16 Circadian Rhythm/ (166802)
17 chronobiology.mp. (7852)
18 (morning or night*).tw,kw. (353070)
19 daytime.mp. or Diurnal*.tw,kw. (149917)
20 bedtime.mp. (23353)
21 (chronotherap* or chrono therap*).tw,kw. (3862)
22 evening.tw,kw. (56243)
23 (breakfast* or supper*).tw,kw. (33205)
24 chronotherapy/ or drug chronotherapy/ (5382)
25 (chronopharmacokinetic* or chronopharmacolog* or chrono pharmaco*).tw,kw. (1951)
26 or/13-25 (637211)
27 12 and 26 (666)
28 exp animals/ not humans/ (17755478)

29 27 not 28 (474)

30 limit 29 to english language (416)

31 30 use medall (145) Medline

32 exp *breast cancer/ (571662)

33 (breast adj2 (cancer or neoplasm* or tumo?r* or carcinoma*)).tw. (771998)

34 32 or 33 (875465)

35 *cancer hormone therapy/ (4582)

36 exp aromatase inhibitor/ or aromatase inhibitor*.tw. (47742)

37 tamoxifen/ (83676)

38 (tamoxifen or anastrozole or exemestane or Fadrozole or formestane or intraovarian peptides or letrozole or plomestane or vorozole).tw. (73976)

39 *"antineoplastic hormone agonists and antagonists"/ (1674)

40 ((hormon* or endocrin* or systemic) adj (treatment or therapy)).tw. (170669)

41 or/35-40 (282483)

42 34 and 41 (103361)

43 circadian rhythm/ (166802)

44 "time of day".tw. (25546)

45 chronobiology.tw. (2801)

46 chronobiology/ (3431)

47 (morning or night*).tw. (351338)

48 (daytime or bedtime).tw. (99406)

49 evening.tw. (55670)

50 (breakfast* or supper*).tw. (33075)

51 chronotherapy/ or chronopharmacology/ (5240)

52 (chronotherap* or chronopharmacokinetic* or chronopharmacolog*).tw. (4599)

53 *time factor/ (3767)

54 or/43-53 (594385)

55 42 and 54 (552)

56 (exp animal/ or nonhuman/) not exp human/ (11945907)

57 55 not 56 (545)

58 limit 57 to english language (492)

59 58 use emczd (309) Embase

60 exp Breast Neoplasms/ (863076)

61 (breast adj2 (cancer or neoplasm* or tumo?r or carcinoma*)).tw. (762057)

62 (breast and (cancer or neoplasm* or tumo?r* or carcinoma*)).kw. (147066)

63 or/60-62 (1032386)

64 exp Aromatase Inhibitors/ (42347)

65 exp Tamoxifen/ (85879)
66 Aromatase Inhibitor*.tw,kw. (21929)
67 (tamoxifen or anastrozole or exemestane or Fadrozole or formestane or intraovarian peptides or letrozole or plomestane or vorozole).tw,kw. (74869)
68 Antineoplastic Agents, Hormonal/tu (10910)
69 ((hormon* or endocrin* or systemic) adj (treatment or therapy)).tw. (170669)
70 or/64-69 (287655)
71 63 and 70 (112917)
72 *Time Factors/ (3708)
73 ("time of day" or time of administration).tw,kw. (34324)
74 Circadian Rhythm/ (166802)
75 chronobiology.mp. (7852)
76 (morning or night*).tw,kw. (353070)
77 daytime.mp. (88880)
78 bedtime.mp. (23353)
79 chronotherapy.tw,kw. (2891)
80 evening.tw,kw. (56243)
81 (breakfast* or supper*).tw,kw. (33205)
82 chronotherapy/ or drug chronotherapy/ (5382)
83 (chronopharmacokinetic* or chronopharmacolog*).tw,kw. (1891)
84 or/72-83 (613734)
85 71 and 84 (664)
86 limit 85 to english language (605)
87 86 use cctr (74) Cochrane
88 31 or 59 or 87 (528)
89 remove duplicates from 88 (361)
90 89 use medall (144) Medline
91 89 use emczd (182) Embase
92 89 use cctr (35) Cochrane
