# Supplementary material for: Does the Time of Day at Which Endocrine Therapy Is Taken Affect Breast Cancer Patient Outcomes?
Source: Curr Oncol. 2021 Jul 6;28(4):2523–8. doi: 10.3390/curroncol28040229 (PMC8293101; doi:10.3390/curroncol28040229)
Supplement: Supplementary file 1 [file curroncol-28-00229-s001.zip › curroncol-1281526-supplementary/Supplemental Figures/Supplemental Figure S2.pdf]

Supplemental Figure S2: Search Strategy 2

Database: Embase Classic+Embase <1947 to 2020 December 31> , Ovid MEDLINE(R) ALL <1946 to December 31, 2020>, EBM Reviews - Cochrane Central Register of Controlled Trials <November 2020>
Search Strategy:
----
1 exp Breast Neoplasms/ (882626)
2 (breast adj2 (cancer or neoplasm* or tumo?r* or carcinoma*)).tw. (793981)
3 (breast and (cancer or neoplasm* or tumo?r* or carcinoma*)).kf. (63717)
4 or/1-3 (1058398)
5 exp Aromatase Inhibitors/ (43237)
6 Aromatase inhibitor*.tw,kw. (22474)
7 exp Tamoxifen/ (87140)
8 (tamoxifen or anastrozole or exemestane or Fadrozole or formestane or intraovarian peptides or letrozole or plomestane or vorozole).tw,kw. (76393)
9 Antineoplastic Agents, Hormonal/tu (11032)
10 ((hormon* or endocrin* or systemic) adj (treatment or therapy)).tw. (176194)
11 or/5-10 (295222)
12 4 and 11 (115402)
13 *Time Factors/ (3935)
14 "time of day".tw,kw. (26413)
15 time of administration.tw,kw. (9006)
16 Circadian Rhythm/ or Circadian.tw,kf. or chronomodulate*.tw,kf. (205621)
17 chronobiology.mp. (7973)
18 (morning or night*).tw,kw. (362566)
19 daytime.mp. or Diurnal*.tw,kw. (153644)
20 bedtime.mp. (24072)
21 (chronotherap* or chrono therap*).tw,kw. (3986)
22 evening.tw,kw. (57606)
23 (breakfast* or supper*).tw,kw. (34164)
24 chronotherapy/ or drug chronotherapy/ (5455)
25 (chronopharmacokinetic* or chronopharmacolog* or chrono pharmaco*).tw,kw. (1973)
26 or/13-25 (676078)
27 12 and 26 (698)
28 exp animals/ not humans/ (18660438)
29 27 not 28 (488)
30 limit 29 to english language (429)
31 30 use medall (155) Medline
32 exp *breast cancer/ (576342)
33 (breast adj2 (cancer or neoplasm* or tumo?r* or carcinoma*)).tw. (793981)
34 32 or 33 (898120)
35 *cancer hormone therapy/ (4696)
36 exp aromatase inhibitor/ or aromatase inhibitor*.tw. (48821)
37 tamoxifen/ (84916)
38 (tamoxifen or anastrozole or exemestane or Fadrozole or formestane or intraovarian peptides or letrozole or plomestane or vorozole).tw. (75479)
39 *"antineoplastic hormone agonists and antagonists"/ (1686)
40 ((hormon* or endocrin* or systemic) adj (treatment or therapy)).tw. (176194)
41 or/35-40 (290004)
42 34 and 41 (105785)
43 circadian rhythm/ (169257)
44 "time of day".tw. (26322)
45 chronobiology.tw. (2842)
46 chronobiology/ (3454)
47 (morning or night* or diurnal*).tw. (408595)

48 (daytime or bedtime).tw. (102306)
49 (evening or circadian or chronomodulate*).tw. (169578)
50 (breakfast* or supper*).tw. (34046)
51 chronotherapy/ or chronopharmacology/ (5313)
52 (chronotherap* or chronopharmacokinetic* or chronopharmacolog*).tw. (4705)
53 *time factor/ (3994)
54 or/43-53 (655791)
55 42 and 54 (591)
56 (exp animal/ or nonhuman/) not exp human/ (12096945)
57 55 not 56 (584)
58 limit 57 to english language (528)
59 58 use emczd (331) Embase
60 exp Breast Neoplasms/ (882626)
61 (breast adj2 (cancer or neoplasm* or tumo?r or carcinoma*)).tw. (783883)
62 (breast and (cancer or neoplasm* or tumo?r* or carcinoma*)).kw. (152567)
63 or/60-62 (1058250)
64 exp Aromatase Inhibitors/ (43237)
65 exp Tamoxifen/ (87140)
66 Aromatase Inhibitor*.tw,kw. (22474)
67 (tamoxifen or anastrozole or exemestane or Fadrozole or formestane or intraovarian peptides or letrozole or plomestane or vorozole).tw,kw. (76393)
68 Antineoplastic Agents, Hormonal/tu (11032)
69 ((hormon* or endocrin* or systemic) adj (treatment or therapy)).tw. (176194)
70 or/64-69 (295222)
71 63 and 70 (115521)
72 *Time Factors/ (3935)
73 ("time of day" or time of administration).tw,kw. (35321)
74 Circadian Rhythm/ (169257)
75 chronobiology.mp. (7973)
76 (morning or night*).tw,kw. (362566)
77 daytime.mp. or diurnal*.tw,kw. (153644)
78 bedtime.mp. (24072)
79 chronotherapy.tw,kw. (2997)
80 evening.tw,kw. (57606)
81 (breakfast* or supper*).tw,kw. (34164)
82 chronotherapy/ or drug chronotherapy/ (5455)
83 (chronopharmacokinetic* or chronopharmacolog*).tw,kw. (1913)
84 (circadian or chronomodulate*).tw,kw. (124315)
85 or/72-84 (676404)
86 71 and 85 (703)
87 limit 86 to english language (641)
88 87 use cctr (71) Cochrane
89 31 or 59 or 88 (557)
90 remove duplicates from 89 (379)
91 90 use medall (155) Medline
92 90 use emczd (193) Embase
93 90 use cctr (31) Cochrane
